# Beyond vision:Cataract and health status in old age, a narrative review

**DOI:** 10.3389/fmed.2023.1110383

**Published:** 2023-03-16

**Authors:** Rita Mencucci, Simone Stefanini, Eleonora Favuzza, Michela Cennamo, Chiara De Vitto, Enrico Mossello

**Affiliations:** ^1^Eye Clinic, Department of Neurosciences, Psychology, Drug Research and Child Health (NEUROFARBA), University of Florence, Florence, Italy; ^2^Division of Geriatric and Intensive Care Medicine, Azienda Ospedaliero Universitaria Careggi, and Department of Experimental and Clinical Medicine, University of Florence, Florence, Italy

**Keywords:** cataract, cataract surgery, functional vision, quality of vision, elderly, frailty, intraocular lenses, accidental falls

## Abstract

Cataract is a leading cause of visual impairment in old age. Lens opacification is notoriously associated with several geriatric conditions, including frailty, fall risk, depression and cognitive impairment. The association is largely attributable to visual impairment, while other mechanisms, associated with extraocular comorbidity and lifestyle, might partly explain this correlation. Available literature suggests that cataract surgery may be effective in decreasing fall risk, improving depressive symptoms and limiting the risk of cognitive impairment and dementia incidence, although intervention studies on these outcomes are still limited. In this review we also emphasize the need to move from the concept of visual acuity to functional vision, especially in the context of the geriatric patient. Research is needed regarding the effect on the cited outcomes of different cataract treatment strategies, such as systematic bilateral versus monolateral surgery and use of different intraocular lenses.

## Introduction

Cataract is the partial or total opacification of the lens, usually progressive and irreversible, leading to loss of vision with medical, social and economic implications. Typically occurring with advancing age, it is a frequent cause of age-related blindness and it is reversible through surgery (1).

It is estimated that 95 million people worldwide are affected by cataract (2). In 2020, the leading worldwide causes of blindness in patients aged 50 years and older were: cataract, followed by glaucoma, under-corrected refractive error, age-related macular degeneration, and diabetic retinopathy (3). To underline the importance of the disease, cataract is responsible for 50% of cases of blindness in middle- and low-income countries, which have poor access to primary care compared to 5% in developed countries (2, 4). The World Health Organization has estimated that with population growth and progressive aging in future years there will be an increase of visual impairment attributable to cataract (5). Furthermore, up to two-thirds of adults with visually significant cataract have been estimated to be undiagnosed, and half of these cases have bilateral visual impairment, often resulting in significant functional impairment (6). Overall, the cited data underline the huge and probably underestimated impact of cataract on visual functioning of older adults. The present narrative review is aimed at assessing the association of cataract with different aspects of health status of aging population, including frailty, falls, fracture, depressive symptoms, and cognitive impairment. Accordingly, we describe potential impact of cataract surgery in old age, and discuss synergies between ophthalmologic and geriatric care possibly resulting in a reduced burden of the disease.

## Cataract, frailty, falls and fractures

### Cataract and frailty

Frailty is defined as a clinical state in which there is an increase in an individual’s susceptibility to developing negative health-related events when exposed to endogenous or exogenous stressors (7). Two main models have been established to define frailty: the *physical frailty phenotype* proposed by Linda Fried and the *deficit accumulation index* elaborated by Kenneth Rockwood (8).

However it is defined, frailty is associated with an increased risk of adverse events, including mortality, disability, and hospital admission. Falls are included among frailty-associated adverse events and, in turn, are associated with fragility fractures, head trauma, disability and mortality risk.

The possible role of cataract as a global frailty biomarker is underlined by data which shows an association with mortality risk (9). Nevertheless, data regarding the association between cataract surgery and mortality are not consistent. In fact, some results suggest a reduced mortality risk after cataract surgery (10), other works show a reduced mortality risk only among the patients who gained better visual acuity (11); on the contrary, some studies suggest a neutral effect after adjusting for confounders (12). Finally, a cohort study has also documented a greater mortality risk among patients that underwent cataract surgery (13).

As a matter of fact, cataract and frailty are both correlated with aging and often coexist. Beyond the parallelism due to demographic factors, an association has been identified between visual impairment and incident physical frailty, independently of coexistent diseases and possible confounders (14); in this work, visual impairment was correlated with a future development of frailty after a 4-year follow-up among non-frail older patients, placing cataract as the most common reversible cause of visual impairment.

On the other hand, a specific association has been detected between cataract and physical frailty in cross-sectional studies, also independently of visual impairment (15), suggesting shared biological mechanisms which include similar age-associated biochemical alterations involving the lens and skeletal muscle protein structures. The cited authors found an association between nuclear cataract in men and a slower gait time (*p* = 0.01) as well as a poorer frailty index score (*p* = 0.01); however cortical and posterior subcapsular cataract in women was correlated with a lower peak expiratory flow rate (*p* < 0.01). Consistently, in a different sample of community-dwelling older patients, there was a significant difference in cataract risk between non-frail (31%), prefrail (37%), and frail groups (42%) (16).

In specific conditions the association between cataract and frailty has known biological explanation. For example, pseudoexfoliation (PEX) syndrome is associated both with an increased risk of nuclear cataract and cataract surgery and with a higher prevalence of cardio- and cerebrovascular disorders, sensorineural hearing loss and Alzheimer-related dementia (17–21). Moreover, posterior subcapsular cataracts may be associated with diabetes mellitus and with steroid treatment, which in turn may correlate with frailty risk independently of lens opacification (22). Other mechanisms, including similar protein aging in lens and in muscle, have been suggested to explain the potential role of cataract as frailty biomarker independently of low vision (15). Further research in this field is needed.

### Cataract and fall risk

Several observational and some randomized studies have examined the association between cataract surgery and fall risk (23–28). Table 1 summarizes the main studies cited in this review.

**Table 1:** Summary of the studies regarding age-related cataract and main health outcomes.

Cataract and frailty							
Study design	First Author	Ref	Country	Publication year	Sample size	Main findings	Limitations
Cross-sectional							
	Klein BEK	15	United States	2006	2,370	Nuclear and cortical cataract in men are significantly associated with a poorer frailty index score, independently of visual acuity and systemic comorbidities. All cataract subtypes are associated with specific frailty indicators.	No standardized frailty measures. Exclusion of subjects with cataract surgery.
	Chen CY	16	Taiwan	2010	2238	Cataract prevalence is significantly different among non-frail, pre-frail and frail subjects.	Subjective assessment of frailty. No multivariate analysis. No significant difference between pre-frail and frail subjects.
Longitudinal							
	Zhu Z	9	China	2020	1,405	Age-related cataract is a predictor of a poorer 10-year survival independently of visual impairment, therefore representing a possible frailty biomarker	No formal frailty assessment. Limited statistical adjustment.
Cataract and falls							
Study design	First Author	Ref	Country	Publication year	Sample size	Main findings	Limitations
Longitudinal							
	Meuleners LB	28	Australia	2014	28,396	Risk of injurious falls is increased between first- and second-eye cataract surgery, and relatively decreased after second-eye surgery	Lack of a control group. Exclusion of subjects with unilateral surgery. Only severe falls recorded.
	Keay L	26	Australia	2022	409	Fall incidence is significantly decreased only after second-eye, not first-eye surgery.	Lack of a control group. Selected sample at enrollment.
Randomized controlled trial							
	Harwood RH	25	United Kingdom	2005	306	First eye cataract surgery reduces 1-year risk of recurrent fall, rate of falling and risk of fractures.	No effect on the risk of a first fall. Selected sample at enrollment.
	Foss AJE	23	United Kingdom	2006	239	Second-eye cataract surgery is followed by a non-significant decrease of 1-year fall rate, in spite of improved visual disability.	Underpowered study. No effect on the risk of a first fall.
Meta-analysis							
	Gutiérrez-Robledo LM	27	Mexico	2021	1,014	First-eye cataract surgery is followed by a decreased fall rate, second-eye surgery does not have significant impact.	Both clinical trials and before-after studies included. Few studies on second-eye surgery. Heterogeneous fall assessment.
Cataract and fractures							
Study design	First Author	Ref	Country	Publication year	Sample size	Main findings	Limitations
Case series							
	Cox A	39	United Kingdom	2005	537	Older subjects with hip fracture have poorer vision than that documented in other older population, with cataract representing the most frequent cause.	Lack of a control group. No multivariate assessment.
Cohort							
	Tseng VL	42	United States	2012	1,113,640	Among older subjects with cataract, surgical treatment is associated with a lower adjusted 1-year hip fracture risk in comparison with no treatment.	Analysis based on administrative data. Treated group is sicker and has a higher absolute risk compared with non-treated, and a higher risk after surgery compared with the year before. No distinction between first-and second-eye surgery.
	Huang H-K	40	Taiwan	2019	115,944	Cataract is independently associated with an increased 6-year risks of osteoporosis and fractures.	Analysis based on administrative data, possible under-diagnosis of osteoporosis.
	Lim J-Y	41	Korea	2022	558,147	Older subjects treated with cataract surgery have a lower 10-year incidence of hip and vertebral fracture than non-treated ones.	No information on non-operated cataract. Analysis based on administrative data. Groups differ for covariates. No distinction between first-and second-eye surgery.
Cataract and depression							
Study design	First Author	Ref	Country	Publication year	Sample size	Main findings	Limitations
Case series							
	Mylona I	55	Greece	2021	150	Greater improvement in visual acuity is associated with greater decrease of depressive symptoms after cataract surgery.	Few subjects with depressive symptoms, mild in severity.
Cross-sectional							
	Kang MJ	47	Korea	2023	4,122	Older subjects with cataract have a greater risk of major depression than those without.	Self-reported diagnosis.
Longitudinal							
	Chen PW	48	Taiwan	2020	233,258	Cataract is associated with increased 7-year risk of incident depression. Within cataract subjects, surgery is associated with a decreased risk of depression compared with no treatment.	Analysis based on administrative data, possible under-recognition of mild depressive symptoms. No distinction between first-and second-eye surgery.
Randomized controlled trial							
	Harwood RH	25	United Kingdom	2005	306	First eye cataract surgery is associated with a decrease of depression and anxiety symptoms.	Psychological status was an ancillary outcome. Selected sample at enrollment.
Cataract and dementia							
Study design	First author	Ref	Country	Publication year	Sample size	Main findings	Limitations
Longitudinal							
	Yu W-K	58	Taiwan	2015	491,226	Among older subjects with cataract, surgery is associated with a decreased 10-year incidence of dementia	Analysis based on administrative data. No distinction between first-and second-eye surgery.
	Lee CS	60	United States	2022	3,038	Among older subjects with cataract or glaucoma, cataract surgery is associated with a decreased 8-year dementia incidence, glaucoma surgery is not.	Ophthalmic diagnoses based on administrative data.
Meta-analysis							
	Kuźma E	51	Germany	2021	6,659	In a meta-analysis of longitudinal, observational studies, cataract is associated with an increased risk of incident dementia.	Not all studies designed to assess dementia incidence. High heterogeneity (sampling, adjustment strategy, exposure and outcome assessment).

Normal aging is accompanied by visual dysfunctions that correlate with fall risk, including reduced visual acuity, reduced contrast sensitivity, reduced depth perception, visual field contraction and prolonged glare recovery (29). Similarly, several specific age-associated ocular diseases have a well-established correlation with recurring fall events, with cataract being both the most prevalent, as previously discussed, and one of the most easily treatable, at least in economically-stable countries.

Vision contributes not only to the detection of tripping hazards on the ground, but also to a patient’s posture and balance through visual-sensory inputs modulated at the cerebellar level, where they are integrated with a proprioceptive signal. Good vision is also associated with high levels of physical activity, thus suggesting its enabling effect on health through the biomechanical benefits of physical exercise on the musculoskeletal system, resulting in a lower fall risk (30). Consistent with these data, a large multinational study has observed a graded association between vision impairment severity and sarcopenia (i.e., loss of muscle mass in old age) (31).

Most literature shows that first-eye cataract surgery reduces the risk of falls in older people, but the effect of second-eye cataract surgery is less clear (25, 27, 32).

Some studies have been summarized in a recent meta-analysis, which documented that fall risk could be reduced by one third after cataract extraction (in comparison with the pre-surgery period), although a significant between-study heterogeneity was observed (27). These data are consistent with the single randomized controlled trial that reported a significant reduction of rate of falls among randomized patients undergoing expedited surgery compared with a postponed-list group, in a 12-month follow-up (25). Moreover, the treatment group showed a better outcome with regard to anxiety, depression and quality of life. Conversely, no significant fall rate decrease was observed after second-eye surgery, both in observational studies and in a single, although underpowered, randomized clinical trial, in spite of a decrease in visual disability and increase in quality of life observed in the latter study (23, 27). Somehow at odds with the cited data, a large Australian observational study found an increase in fall rate during the 2 years following first-eye cataract surgery, while waiting for the second-eye surgery, with a relative decrease only after the treatment of the second eye (28). These data are consistent with a US population-based study showing that, after a 2-year follow-up, older patients undergoing monolateral cataract surgery had a greater decline in motor function in comparison with a general older population without severe visual impairment, while this decline was not observed in the subgroup undergoing bilateral surgery (33). A recent cohort Australian study on patients referred for bilateral cataract surgery, confirmed an absolute decrease in the fall rate only after the second intervention (26). This is consistent with data that show an association between stereopsis and fall risk, which is even more important than that observed for visual acuity, thus suggesting that good binocular vision, which can be attained with bilateral surgery, may be needed to minimize fall risk (23, 34). On the whole, cited data suggest that cataract is a marker of increased risk of motor impairment and fall risk, possibly beyond vision impairment, and that a bilateral correction is probably needed to achieve a substantial risk reduction, while patients undergoing monolateral surgery may show a paradoxical increase in fall risk.

Yet more research is needed to support a systematic policy of bilateral surgery to decrease fall risk in an aging population (35).

### Cataract and hip fractures

Approximately one in three community-dwelling individuals over the age of 65 reports at least one fall event per year, with this risk proportionally increasing with age, determining in 5–10% of cases fragility fractures and 1–2% of cases hip fractures, also due to the frequent coexistence of osteoporosis (36).

Nevertheless, the role of osteoporosis over the years has been downplayed. Siris et al., using data from NORA (National Osteoporosis Risk Assessment), examined a population of almost 150.000 white, postmenopausal women aged 50 to 104 years (mean age 64.5 years) and observed that 82% of postmenopausal women with fractures had T-scores higher than −2.5, the threshold value below which osteoporosis is diagnosed (37). This makes it clear that reduced bone mineral density (BMD) is not actually as decisive as we might think in hip fracture development. The most recent literature points out that BMD and risk of falling independently increase fracture risk, with a need for multifactorial interventions for primary and secondary prevention of fragility fractures (36).

One area for further study is represented by the relationship between visual impairment, including cataract and fractures, with the aim of identifying effective prevention strategies. It has been clearly demonstrated that blindness (defined as a best corrected visual acuity ≤20/500 in the better eye) increases the risk of hip and vertebral fractures (38). Among conditions of visual impairment, untreated cataract has been identified as the main cause of hip fracture in a UK sample (39). In a Taiwanese matched cohort study cataract was associated with an increased risk of hip and vertebral fractures over a 6.4-year follow-up (40). To note, patients with cataract had a greater baseline comorbidity and an increased risk of osteoporosis incidence during follow-up, suggesting it may represent a frailer population, beyond visual impairment (40). Little data, and no specifically designed intervention studies, are available regarding the association between cataract surgery and hip fractures. In the previously cited Taiwanese study, patients undergoing cataract surgery showed a decreased fracture risk in comparison with those with non-operated cataract. A recent nationwide Korean cohort study confirmed that older patients who underwent cataract surgery showed a lower incidence of hip and vertebral fragility fractures than those who did not (41). In a previous Medicare cohort study, patients that underwent surgery in comparison with patients with non-operated cataract had similar hip fracture rates in a 12-month follow-up period. However, patients in the surgery group were older, had more severe comorbidity and disability and were more frequently affected by severe cataract and, after adjusting for these covariates, hip fracture rate was significantly lower in the surgery group, with an absolute risk difference of about 0.2% per year, and more beneficial effects observed among older patients, more advanced cataract and greater comorbidity (42). Overall, these studies suggest that cataract surgery in elderly patients may reduce and prevent the incidence of hip and vertebral fragility fractures. No study has compared fracture risk associated with first- and second-eye cataract surgery.

### Cataract, depression and cognitive impairment

Depression is common in old age and is typically associated with chronic disease and multimorbidity, psychosocial adversity, cognitive impairment and disability (43).

Several studies have shown an association between depression in old age and visual disturbances, including cataract. The association between low vision and depression may be explained by reduction in daily activity, such as reading, loss of autonomy, difficult social interaction and loss of self-esteem (44). A recent metanalysis has identified a huge 25% prevalence of depression in samples of patients referred to eye clinics and low vision rehabilitation centers. The prevalence of depression was even higher, estimated as 33%, in the subgroup of studies that did not adopt exclusion criteria, and dropped to 18% when patients with comorbidity, mainly cognitive impairment, were excluded (45). A French cohort study has shown that patients with low vision have a threefold increase of depression risk in a 10-year follow-up, but that patients with depression have a 60% increased risk of vision impairment incidence (46). Regarding the specific effect of cataract, its diagnosis has been recently associated with a 65% increase of major depression risk in the cross-sectional analysis of a representative sample of older Korean citizens (47). Moreover, cataract was specifically associated with a 78% increase of depression risk after a 7.8-year follow-up in a propensity score matched cohort study in Taiwan (48).

Several studies have recently examined the association between vision impairment and risk of cognitive decline. A recent metanalysis has observed an association of low vision with an increased risk of cognitive impairment and dementia incidence (49). A dose–response association has been observed in a large UK cohort, with dementia risk being greatest among patients with severe vision impairment (50). In a meta-analysis that compared the risk of cognitive impairment between different causes of low vision, cataract and diabetic retinopathy were associated with an increased risk of dementia and Alzheimer’s disease (51).

Regarding cataract surgery, some studies suggest that it can improve depressive symptoms and anxiety and may be associated with a decreased risk of cognitive impairment (52–54). In a previously cited cohort study, subjects with cataract undergoing surgery had a 25% less depression risk in comparison with untreated ones over a 7.8-year follow-up (48). Moreover, data from the previously-cited randomized controlled study on expedited cataract surgery have shown a significant decrease in depression and anxiety in the early treatment group (25). Of notice, an association has been observed between visual acuity improvement and depressive symptoms decrease after phacoemulsification, thus highlighting the importance of successful surgery for this specific outcome (55).

Data have been less consistent over the years regarding the association between cataract surgery and risk of cognitive impairment, as older studies reported no significant effects on neuropsychological functions (56, 57), while more recent ones showed a protective association on cognitive impairment and dementia risk (58). A small study conducted using functional magnetic resonance imaging suggested functional and morpho-structural improvements in visual and cognitive-related brain areas after cataract surgery (59). In a large US cohort including older patients (mean age 74) with a diagnosis of cataract and glaucoma, cataract surgery was independently associated with a significant decrease in dementia risk in a 7.8-year follow-up, while glaucoma surgery was not (60).

A recent systematic review and meta-analysis focusing on psychiatric and neuropsychological assessments provided further evidence that cataract surgery has a positive effect not only on depressive symptoms but also on cognitive function in older patient (61).

## Discussion

Visual acuity is the main parameter evaluated in ophthalmology to monitor visual progress of a medical or surgical treatment. The term “visual acuity” refers to the ability of the human eye to detect and perceive the smallest details of an object at a given distance (62, 63). Normal visual acuity depends on the transparency of the eye’s dioptric media, the correction of any refractive defect, and the integrity of the macula and optic pathways. However, it is now well established that visual acuity provides only raw data on the overall functioning of sight, indeed it does not consider a patient’s ability to use his or her visual apparatus within a complex and dynamic socio-cultural environment (64, 65). The visual acuity test with Snellen tables is a high-contrast test: recognizing black letters on a white background allows even a patient with low contrast sensitivity to achieve 20/20 (66). Moreover, high visual acuity can be found in patients with severe peripheral visual field deficit: despite high performance using Snellen’s table, a patient with visual field defect may have difficulty relating to the outside world and is potentially limited in a large number of daily activities, implying reduced quality of life and poor social and occupational functioning (67).

Concerning the cataract patient, visual acuity is used to address surgical indication. In Europe, for example, it is customary to advise patients with visual acuity of 6/12 or less in one or both eyes to undergo surgery (68). However, this advice has clear limits: a patient with preserved visual acuity but affected by a posterior subcapsular cataract may experience bothersome nighttime glare at the sight of traffic lights, therefore for a nighttime driver, even with high VA, a subcapsular cataract can severely limit his or her functioning and merit expedited surgery. Indeed, the National Institute of Health and Care Excellence guidelines for the management of cataracts established that the assessment of visual acuity as an indication for cataract surgery fails to recognize other visual impairments that may limit the activities of daily living and hence require intervention (69).

Nevertheless, cataract surgery can have intraoperative or postoperative complications, such as endophthalmitis, posterior capsular ruptures, IOL (intra-ocular lens) dislocations, refractive errors, endothelial damage and dry eye (70–73). Even though nowadays these complications are rare, they can affect the postoperative visual outcomes and quality of life. Therefore, the patients should be informed of these risks, but it has to be pointed out that the benefits of surgery very often overcome the possible complications in visually-impaired individuals.

It is also necessary to consider the visual system as binocular. Precise correction of 1 eye by an IOL (intra ocular lens) while waiting for the contralateral eye to be treated could lead to non-negligible anisometropia and sometimes diplopia. This phenomenon could partly explain the increased incidence of falls and fractures after cataract surgery that is documented in some studies, although it cannot be ruled out that phacoemulsification induces patients to consider themselves freer, thus leading them to perform more activities and expose themselves to the risk of falling.

Regarding fall risk, it is reported that multiple “fallers” usually have decreased vision, as indicated by all visual tests, with impaired depth perception, contrast sensitivity and low-contrast visual acuity being the strongest risk factors (74).

There are a few older studies in literature that have found that visual impairment is not a predictor for the risk of falling in old age (75–78). However, most of these studies only assessed a limited aspect of the global visual functioning, that it to say visual acuity. Other studies showed a lack of association between fracture risk and visual impairment when only visual acuity was evaluated (79).

Lastly, patients’ necessities in relation to his or her daily activities must be taken into account when choosing which IOL to implant. For example, a classic monofocal IOL may provide perfect distance visual acuity but limit the range of action at intermediate and close distances. In the context of fall risk and femur fracture, the intermediate distance is perhaps the most impactful. Recognizing an obstacle requires good contrast sensitivity (especially at night), sense of depth, color perception, motion perception, good visual processing speed as well as an optimal binocular field of view. All these factors fall under the concept of *functional vision,* and should be evaluated synergistically to develop a “cataract frailty index” that could select patients at risk of falling, on whom preventive action can be taken with tailored surgical strategies (80).

Comparison of different intraocular lenses regarding visual impairment, visual function and patient satisfaction are becoming available (81, 82). Similar studies addressing outcomes which are specifically relevant to older populations, such as fall incidence, depressive symptoms and cognitive impairment, are needed to guide clinicians’ choice.

To summarize what has been said so far, cataracts increase the risk of developing frailty, falls, fractures, depression and cognitive impairment, and reduce the percentage of functional reserve of an individual over time. *Functional reserve* refers to a patient’s residual capacity to perform his or her physiological activities (83). It is conceivable that by assessing functional vision instead of visual acuity, surgery should be planned within an early “window of opportunity” to prevent the aforementioned geriatric adverse events.

A limitation of our review is the narrative design: further systematic reviews are necessary in order to better describe the current knowledge on the different aspects of this topic.

## Conclusion

Cataract is a primary cause of visual impairment worldwide and, among older subjects, is associated with frailty, fall risk, depressive symptoms, and neurocognitive decline. Due to the high prevalence and the frequent lack of recognition of lens opacification, a systematic screening of visual impairment with a timely referral to the ophthalmologist is advised to prevent progression to bilateral visual impairment and possibly prevent negative health outcomes. In particular, it is necessary to include visual performance in comprehensive geriatric assessment, in order to identify subjects at risk and to develop questionnaires or clinical indices to assess the impact of *functional vision* on daily activities. The introduction of visual assessment in geriatric clinical practice would allow an appropriate referral to the ophthalmologists, with the aim to decide, with a greater clinical awareness, whether cataract extraction is indicated.

On the other hand, ophthalmologists should adopt a comprehensive approach to older subjects, keeping a focus on individual priorities, global autonomy and cognitive difficulties, and tailoring the IOL choice beyond visual function. Indication for surgery should be considered in relation not only to *visual acuity*, but also to the *functional vision* assessment, daily needs and individual priorities. A geriatric referral may be helpful for the ophthalmologist to decide regarding surgery in complex cases, including those with cognitive decline and multimorbidity. After surgery a joint geriatric and ophthalmologic follow-up may allow the assessment of treatment effects on different domains of health status, and may possibly help decision regarding second-eye surgery in frail older subjects.

Generally speaking, cataract surgery should be encouraged for both visual recovery and prevention of negative health-related events in frail patients or those with neurocognitive impairment. However, several research areas remain to be addressed with the aim of identifying the most effective strategies to reduce the global health impact of cataract. In particular, it is conceivable that the protective effect of cataract treatment on functional impairment, falls incidence and dementia risk may be time-dependent, with the need to identify a “window of opportunity” for surgery, before the frailty process becomes irreversible. Therefore, valid and easy-to-use screening instruments of visual impairment, focusing on the impact on daily activities, are needed in primary care and routine geriatric practice. Moreover, the specific role of different intervention strategies, such as systematic bilateral versus unilateral surgery or use of different intraocular lenses, deserves further studies. Most important, future intervention studies should increasingly include global health outcomes, such as disability and quality of life, falls and fracture incidence, depressive symptoms and cognitive impairment.

## Author contributions

RM, SS, and EM wrote the first draft of the manuscript. EF, MC, and CV wrote sections of the manuscript. All authors contributed to conception, design of the review, manuscript revision, read, and approved the submitted version.

## Conflict of interest

The authors declare that the research was conducted in the absence of any commercial or financial relationships that could be construed as a potential conflict of interest.

## Publisher’s note

All claims expressed in this article are solely those of the authors and do not necessarily represent those of their affiliated organizations, or those of the publisher, the editors and the reviewers. Any product that may be evaluated in this article, or claim that may be made by its manufacturer, is not guaranteed or endorsed by the publisher.
